# Longitudinal evaluation of intra-patient changes in computed tomography-based body composition measures

**DOI:** 10.1007/s00261-026-05410-7

**Published:** 2026-03-05

**Authors:** B. Dustin Pooler, John W. Garrett, Matthew H. Lee, Ronald M. Summers, Perry J. Pickhardt

**Affiliations:** 1 School of Medicine and Public Health, University of Wisconsin–Madison, Madison, USA; 2 Imaging Biomarkers and Computer-aided Diagnosis Laboratory, Department of Radiology and Imaging Sciences, National Institutes of Health Clinical Center, Bethesda, USA

**Keywords:** Artificial intelligence (AI), Body composition, Computed tomography (CT), Abdomen

## Abstract

**Objective:**

To report longitudinal intra-patient changes in CT-based body composition using fully automated AI tools in an adult patient sample.

**Methods:**

This retrospective longitudinal study included 15,616 adult patients (mean age at first CT, 53.0 ± 14.7 years; 7096 male, 8520 female) who underwent at least two abdominal CT examinations at least five years apart (mean study interval, 9.1 ± 3.3 years, range, 5.0-20.4 years) at a single academic institution between January 1, 2000 and February 28, 2021. CT examinations were not restricted based on patient setting, clinical indication, or IV contrast media use. Seven fully automated AI body composition tools quantifying vertebral trabecular attenuation, skeletal muscle area and attenuation, visceral adipose tissue (VAT) area and attenuation, subcutaneous adipose tissue (SAT) area, and VAT/SAT ratio (VSR) were applied to each patient’s first and last available abdominal CT. Change in body composition per year were determined using the first and last available CT scans. T-test and linear regression were used to assess sex and age as predictors of longitudinal body composition change, respectively.

**Results:**

Significant differences in sex-specific mean rates of change were observed for all measures (*p* < 0.05) except muscle attenuation. Certain CT biomarkers showed varying rates of change among younger, middle-age, and older adults. Age significantly predicted body composition measures except VSR in female patients, although effect size was small (*R*^2^ values 0.002–0.040).

**Conclusion:**

There are significant age and sex-specific differences in longitudinal, intra-patient body composition changes over time.

## Introduction

Measures of body composition, including bone mineral density, abdominal wall musculature, and abdominal fat are being increasingly recognized as important predictors of health outcomes [1–5]. The advent of automated artificial intelligence (AI) algorithms enabling opportunistic acquisition of these measures from abdominal CT examinations has allowed for deployment of these tools on population-scale patient samples, some in excess of 100,000 patients [6], with the ultimate goal of real-time clinical use for risk assessment and triage to appropriate preventative services. Research efforts leveraging these large patient samples have begun the process of establishing normative values for many of these body composition measures [6, 7] and have been used in the development of biological age models [8].

Prior work has shown that body composition measures differ by sex and change with age [6–9]. Trends in body composition across the age spectrum vary by specific measure. For example, average vertebral trabecular and abdominal muscle density decrease with age in a relatively linear fashion while average visceral abdominal fat area increases into middle age before decreasing in old age [6]. These average values and trends may also be affected by factors like systemic disease and other comorbid conditions.

Critically, previously reported changes in body composition with age in larger patient samples have been derived from cross-sectional data [6, 7]. While this method is important in establishing population-scale normative values and trends, it does not yield any information about changes in body composition over time within individual patients. Consider a patient who is above average in a certain measure at one point in time and then later found to be below average in that same measure: the change over time is arguably more relevant to the health of the patient than where that patient was relative to average at either time point.

Prior studies have assessed intra-patient changes in body composition following specific weight loss interventions, such as bariatric surgery [10] or semaglutide [11], or in patients with specific diseases such as Crohn’s [12], using both imaging based and non-imaging based measures. However, currently there is little in the published literature describing interval changes in CT-based body composition measures within a more general patient group not undergoing targeted therapy [9]. The aim of this study is to report longitudinal, intra-patient changes in CT-based body composition and assess for age and sex-specific differences in rates of body composition change using fully automated AI body composition tools in adult patients undergoing abdominal CT.

## Methods

This retrospective longitudinal study was conducted at a single academic institution (University of Wisconsin School of Medicine and Public Health). The study protocol was HIPAA-compliant and approved by the institutional review board. The requirement for written informed consent from patients was waived.

### Patient sample

An electronic search of the institutional PACS was conducted for adult (age ≥ 18) patients who had at least two abdominal CT examinations at least 5 years apart between January 1, 2000, and February 28, 2021. CT examinations were not restricted by indication and included those performed in inpatient, outpatient, urgent care, and emergency settings. Examinations included those performed with or without IV contrast media; examinations of the abdomen and pelvis; chest, abdomen, and pelvis; chest and abdomen, or abdomen alone (hereafter, all described as abdominal CT examinations). This initial search yielded 17,662 unique patients. For each patient, the first CT examination chosen was the first CT available that met inclusion criteria. For patients who had multiple additional CT examinations at least five years later, the final available examination was chosen for inclusion. For examinations that included more than one axial series, a thin-section axial series (i.e., slice thickness of 1.25 mm, reconstructed at 0.625-mm interval) was preferentially selected for assessment by the AI tools; nonetheless, the AI body composition tools function using thin- or thick-section data [13]. In either case, the AI tools perform slice thickness homogenization as described later in the methods.

CT examinations were excluded if they were not performed with the patient in supine position (e.g., patient in prone or decubitus position), as determined by DICOM header data or a body part regression function within the AI tools (as described later in Methods); if the examination’s anatomic coverage was inadequate (i.e., did not include the top of the diaphragm through the bottom of the kidneys as well as vertebral levels L1 through L3), as determined by a body part regression function within the AI tools; or if other technical factors precluded AI tool deployment (e.g., limited FOV or excessive artifact). These exclusion criteria were assessed collectively using automated processes, and the individual numbers of examinations excluded for each reason were not tracked; the examinations were not manually reviewed due to the large number of examinations in the data set. The exclusion criteria and the processes for their assessment were similar to those used in prior work [6, 14].

Following the exclusions, the final study sample comprised 15,616 patients, including 7096 males (mean [± SD] age, 54.1 ± 14.3 years; median age, 55 years; age rage, 18–95 years) and 8520 females (mean [± SD] age 52.0 ± 14.9 years; median age, 53 years; age range 18–93 years). Each patient had two CT examinations (initial and final) included in the study. Mean [± SD] interval between CT examinations was 9.1 ± 3.3 years with a median of 8.4 years and range of 5.0–20.4.0.4 years. Patient demographic characteristics are listed in Table 1.

**Table 1:** Patient demographic characteristics

Variable	All (*N* = 15,616)	Male (*N* = 7096)	Female (*N* = 8520)
Age (y)			
Mean (± SD)	53.0 ± 14.7	54.1 ± 14.3	52.0 ± 14.9
Median	54	55	53
Range	18–95	18–95	18–93
Race (N, %)			
White	14,285 (91.5)	6532 (92.1)	7753 (91.0)
Black or African American	798 (5.1)	348 (4.9)	450 (5.3)
Asian	262 (1.7)	104 (1.5)	158 (1.9)
American Indian or Alaska Native	153 (1.0)	56 (0.8)	97 (1.1)
Native Hawaiian or Pacific Islander	16 (0.1)	7 (0.1)	9 (0.1)
Unknown or no response	102 (0.7)	49 (0.7)	53 (0.6)
Ethnicity (N, %)			
Not Hispanic or Latino	15,125 (96.9)	6915 (97.4)	8210 (96.4)
Hispanic/Latino	427 (2.7)	147 (2.1)	280 (3.3)
Unknown or no response	64 (0.4)	34 (0.5)	30 (0.4)

### Description of body composition tools

The AI body composition tools used in this study have been trained on approximately 44,735 cases across two institutions, the University of Wisconsin School of Medicine and Public Health and the NIH. The methodology for the complete AI processing pipeline for the entire suite of explainable CT-based cardiometabolic biomarkers has been previously described in detail [15]. A commercial license for the pipeline’s clinical application has been provided to multiple entities. The tools have been validated by numerous prior works [8, 9, 14, 16–24], including at institutions external to those used for training [14, 22]. The tools are packaged into a single fully automated container for processing an inputted CT series. The tools function independently of one another; the adequacy or failure of one tool is not dependent on the performance of the others. The tools initially perform preprocessing to normalize the slice thickness to 3 mm (with 3-mm spacing) and correct for offsets in CT attenuation values. The tools then apply a body part regression function to determine if the series has adequate anatomic coverage and to provide slice positions for relevant anatomic landmarks [25, 26].

The tools then perform automatic segmentation of tissues to produce the following measurements: trabecular bone attenuation (in Hounsfield units) of the L1 vertebral body; cross-sectional area (in cm^2^) and attenuation (HU) of abdominal muscle at L3; cross-sectional area and attenuation of visceral adipose tissue (VAT); cross-sectional area of subcutaneous adipose tissue (SAT) at L3. A VAT/SAT ratio (VSR) is also calculated. The AI tools were deployed on the initial and final CT examinations for each of the 15,616 patients, and the output values were recorded. As a part of the output process, segmentation maps are produced for each tool and can be used for quality assurance purposes. Table 2 lists valid output reference ranges for each of the tools, as determined by prior studies [17, 18, 21, 27]. Prior studies have shown that increases in CT attenuation with the administration of iodinated intravenous contrast are small and predictable for L1 trabecular bone, L3 muscle, and L3 VAT attenuation [23, 28], with previously determined adjustment factors applied for non-contrast examinations to allow pooled evaluation of contrast-enhanced and unenhanced examinations [6, 28].

**Table 2:** AI tool reference ranges

AI Tool (units)	Minimum	Maximum
L1 vertebral body attenuation (HU)	−50	1200
L3 muscle attenuation (HU)	−50	200
L3 muscle area (cm^2^)	25	500
L3 VAT attenuation (HU)	−120	−30
L3 VAT area (cm^2^)	0	1200
L3 SAT area (cm^2^)	0.1	1000.0
VAT/SAT ratio	0.0	5.0

AI Artificial intelligence, VAT Visceral adipose tissue, SAT Subcutaneous adipose tissue

### Data and statistical analysis

Output values for each of the AI body composition biomarkers were recorded for the initial and final CT examinations of each patient. Rates of change for each of the tool outputs were calculated for each patient and were divided by the study interval to produce an annualized rate of change. The independent samples t-test was used to assess differences in rate of change between sexes. A linear regression model was used to evaluate age at initial CT as an independent predictor of each of the seven body composition measures. Missing data were left blank in the regression analyses, without imputation.

A *p* value of 0.05 was used as the criterion for statistical significance. All descriptive statistics and analyses were performed using Microsoft Excel (Version 2408, Build 17928.20538) and SPSS (Version 28.0.1.0).

## Results

Mean initial and final values and annualized rates of change for each of the seven AI body composition measures by sex are summarized in Table 3. In general, for both males and females, vertebral attenuation, muscle attenuation, and muscle area decreased over time and VAT attenuation, VAT and SAT area, and VSR increased over time. There were statistically significant differences in rate of change between males and females for all AI body composition measures (*p*-value range, < 0.001–0.031) except L3 muscle attenuation (mean change −1.03 HU/year for males, −0.99 HU/year for females, *p* = 0.110).

**Table 3:** Mean initial and final values and body composition measures by sex

Body Composition Measure	Men (*N* = 7096)			Women (*N* = 8520)			*P*
	Initial	Final	Annual Change	Initial	Final	Annual Change	
L1 vertebra attenuation* (HU) [± SD]	160 [48]	146 [56]	−1.45 [6.27]	170 [52]	152 [66]	−1.80 [6.80]	< 0.001
L3 muscle attenuation* (HU) [± SD]	34 [14]	25 [17]	−1.03 [1.43]	29 [16]	20 [18]	−0.99 [1.36]	0.110
L3 muscle area (cm^2^) [± SD])	183 [36]	176 [39]	−0.79 [3.77]	130 [28]	129 [31]	−0.14 [2.67]	< 0.001
L3 VAT attenuation* (HU) [± SD]	−93 [11]	−91 [13]	0.11 [1.35]	−90 [12]	−89 [14]	0.06 [1.26]	0.021
L3 VAT area (cm^2^) [± SD]	218 [130]	245 [142]	3.07 [13.08]	126 [101]	146 [106]	2.11 [9.17]	< 0.001
L3 SAT area (cm^2^) [± SD]	194 [106]	203 [113]	0.99 [10.08]	258 [137]	271 [150]	1.39 [13.08]	0.031
L3 VAT/SAT ratio [± SD]	1.18 [0.69]	1.29 [0.70]	0.012 [0.645]	0.50 [0.38]	0.57 [0.40]	0.008 [0.042]	< 0.001

*Adjusted for IV contrast media

VAT visceral adipose tissue, SAT subcutaneous adipose tissue

**Fig. 1:**
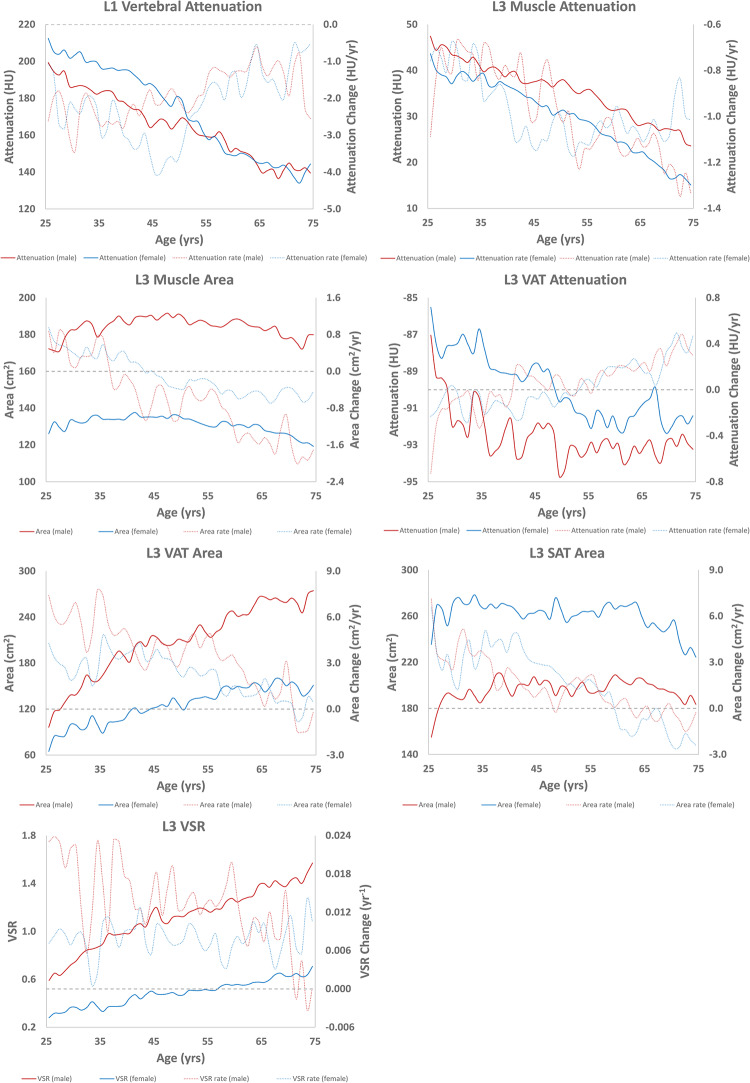
Mean body composition values and annualized rate of change by age and sex. Mean initial body composition values by age for males (solid red) and females (solid blue) with associated annualized rate of change (males, dotted red; females, dotted blue) for L1 vertebral attenuation (top row left), L3 muscle attenuation (top row right), L3 muscle area (second row left), L3 visceral adipose tissue (VAT) area (second row right), L3 VAT area (third row left), L3 superficial adipose tissue (SAT) area (third row right) and VAT/SAT ratio (VSR, bottom row left). The scales for mean body composition values are presented on the left vertical axes and the scales for rates of change are presented on the right vertical axes. For rate of change, zero is denoted as a dashed gray line where applicable. These graphs highlight several important trends. There is a pronounced trough in rate of change of vertebral attenuation for females between ages 45 and 55, indicating accelerated bone density loss during these years. For females, the most rapid decrease in muscle attenuation occurs between the ages of 40 and 50 but occurs between 50 and 60 for males. Both sexes experience an increase in muscle area in early adulthood with a plateau in middle age and a decrease in old age. VAT area increases with age for both sexes, although the rate of increase slows with age. In contrast, SAT area increases most rapidly in early adulthood but plateaus in middle age and decreases in old age

**Fig. 2:**
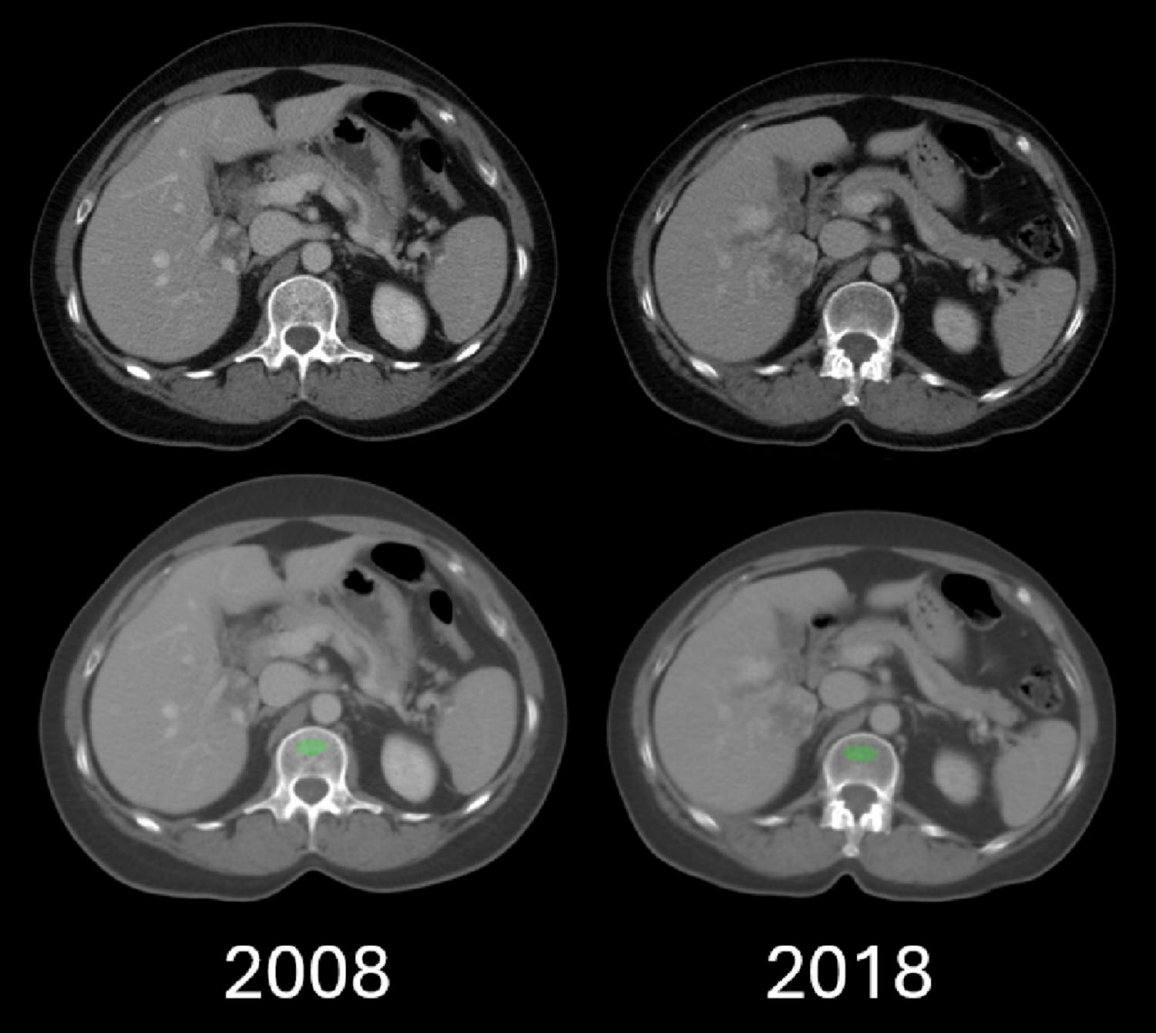
Change in L1 vertebral body attenuation over a 10-year interval. Change in L1 vertebral body attenuation in a 53-year-old female who underwent CT of abdomen with IV contrast media in 2008 (left column) and again in 2018 (right column) at age 64. Axial slices are shown at the L1 level for both time points, including original CT images (top row) and images showing the fully automated segmentation of L1 vertebral body trabecular bone (green). A decrease in L1 trabecular bone attenuation between 2008 and 2018 is visually apparent. AI-measured L1 vertebral body attenuation decreased from 166 Hounsfield units (HU) to 136 HU, for a net decrease of −30 HU over a 10.4-year interval and a rate of −2.9 HU/year. At both time points, the CT indication was to evaluate an incidentally discovered giant hemangioma, partially visible in the medial right lobe of the liver

**Fig. 3:**
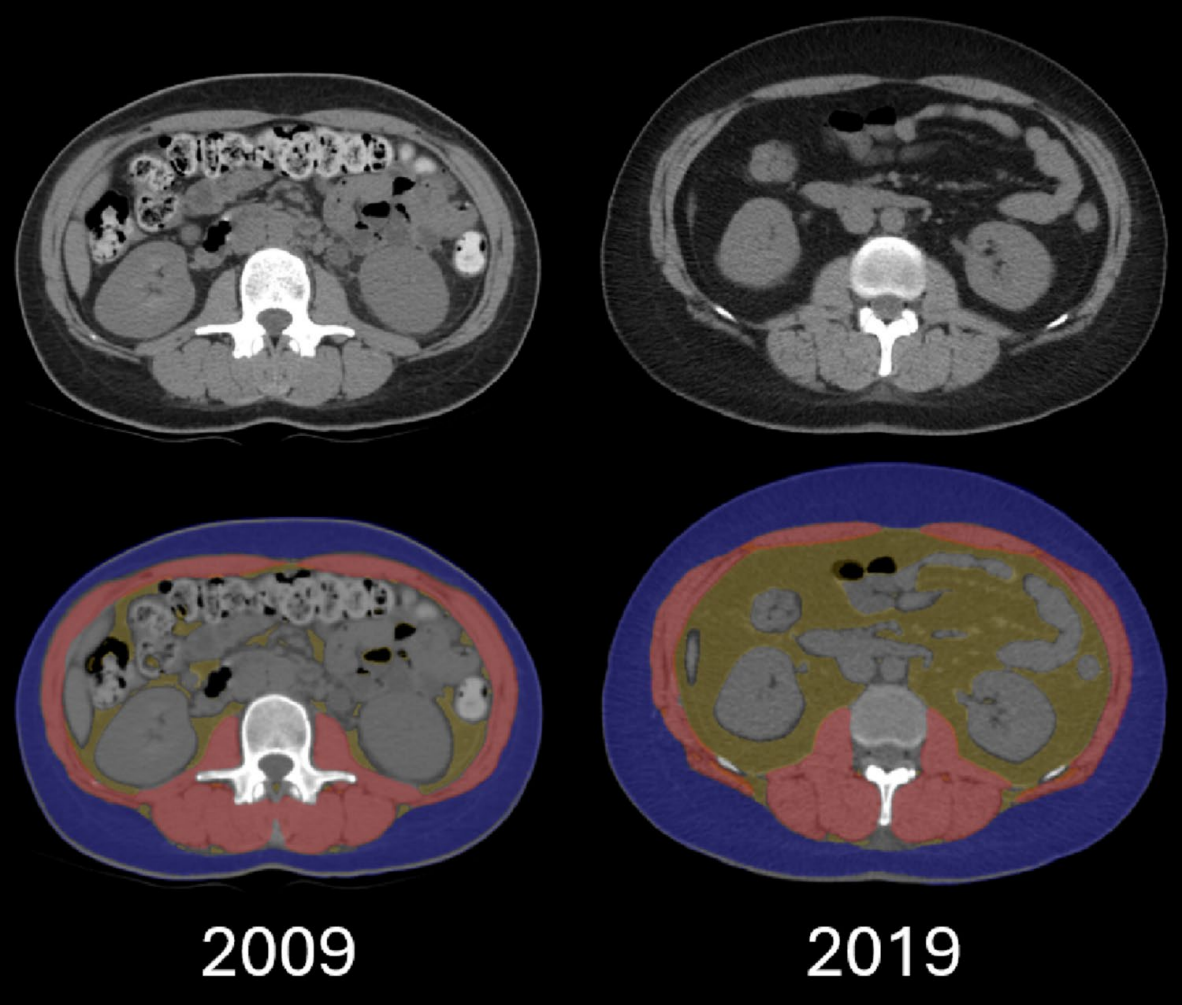
Change in skeletal muscle and abdominal adipose tissue over a 10-year interval. Change in skeletal muscle and abdominal adipose tissue in a 36-year-old female who underwent non-contrast CT of abdomen in 2009 (left column) and again in 2019 (right column) at age 47. Axial slices are shown at the L3 level for both time points, including original CT images (top row) and images showing the fully automated segmentation of skeletal muscle (red), visceral adipose tissue (VAT, yellow), and subcutaneous adipose tissue (SAT, blue). There are visually apparent increases in VAT and SAT area with subtler apparent decreases muscle and VAT density. Change in muscle area is more difficult to assess visually but appears similar. AI-measured skeletal muscle density decreased from 45 Hounsfield units (HU) to 28 HU, for a net decrease of −17 HU over a 10.5-year interval and a rate of −1.7 HU/year. AI-measured skeletal muscle area increased from 110 cm^2^ to 142 cm^2^, for a net increase of 32 cm^2^ and a rate of 3.1 cm^2^/year. AI-measured VAT density decreased from −68 HU to −95 HU, for a net decrease of −27 HU and a rate of −2.7 HU/year. AI-measured VAT area increased from 38 cm^2^ to 211 cm^2^, for a net increase of 173 cm^2^ and a rate of 16.5 cm^2^/year. AI-measured SAT area increased from 113 cm^2^ to 316 cm^2^, for a net increase of 203 cm^2^ and a rate of 19.4 cm^2^/year. Calculated VAT-to-SAT ratio (VSR) increased from 0.34 to 0.67, for a net increase of 0.33 and a rate of 0.03/year. At both time points, the CT indication was to evaluate for kidney stones in the setting of flank pain

Mean annualized rates of change for each of the body composition measures by age at initial CT (reported in age groups of 18–39, 40–59, and 60 + years) and associated linear regression statistics are summarized in Table 4 and mean initial body composition values with associated rates of change by age and sex are displayed in Fig. 1. Age at initial CT was an independent predictor for annualized rate of change for all body composition measures in males and all body composition measures except VSR in females, with some notable trends. Although both sexes had decreased L1 vertebral body attenuation with age, the rate of decrease was faster for males in early adulthood (−2.33, −1.70, and −1.12 HU/year for age groups 18–39, 40–59, and 60 + years, respectively) and faster for females in middle age (−1.84, −2.48, −0.73 HU/year) (Fig. 2). Decreases in skeletal muscle attenuation occurred more rapidly with age in males (−0.75, −0.99, and −1.19 HU/year) but appeared to plateau after middle age in females (−0.78, −1.08, −1.00 HU/year) (Fig. 3). Younger adults of both sexes showed an increase in skeletal muscle area (positive rate of change) while middle-aged (age 40–59) and older (age 60+) adults of both sexes showed a decrease in muscle area, with a more rapid decrease in males (0.47, −0.61, −1.57 cm^2^/year for males; 0.57, −0.20, −0.53 for females). Both sexes experienced increases in VAT area across the age spectrum, but the rate of increase slowed with age (5.96, 3.97, 0.62 cm^2^/year for males; 3.31, 2.67, 0.44 cm^2^/year for females). Similarly, the rate of SAT area change decreased across the age spectrum, becoming negative (decrease in SAT area) in older age (3.79, 1.27, −0.57 cm^2^/year for males; 4.55, 2.17, −1.90 cm^2^/year for females). Males experienced a slower increase in VSR with age (0.018, 0.014, and 0.007/year) but for females this rate of increase remained relatively constant with age (0.007, 0.008, 0.008/year).

**Table 4:** Mean annualized rate of change of body composition measures by age at initial CT and sex

Body Composition Measure	Age Group (y)			Regression Statistic	
	18–39 (*N* = 1098)	40–59 (*N* = 3421)	60+ (*N* = 2577)	*P*	R^2^
Male Patients (*N* = 7096)					
L1 vertebra attenuation* (ΔHU/yr [± SD])	−2.33 [6.50]	−1.70 [6.44]	−1.12 [7.97]	< 0.001	0.005
L3 muscle attenuation* (ΔHU/yr [± SD])	−0.75 [1.20]	−0.99 [1.46]	−1.19 [1.45]	< 0.001	0.014
L3 muscle area (Δcm^2^/yr [± SD])	0.47 [3.41]	−0.61 [3.77]	−1.57 [3.73]	< 0.001	0.033
L3 VAT attenuation* (ΔHU [± SD])	−0.21 [1.32]	0.06 [1.33]	0.30 [1.37]	< 0.001	0.020
L3 VAT area (Δcm^2^/yr [± SD])	5.96 [10.65]	3.97 [13.09]	0.62 [13.59]	< 0.001	0.024
L3 SAT area (Δcm^2^/yr [± SD])	3.79 [11.13]	1.27 [9.96]	−0.57 [9.45]	< 0.001	0.024
L3 VAT/SAT ratio (Δ/yr [± SD])	0.018 [0.054]	0.014 [0.063]	0.007 [0.071]	< 0.001	0.004
Female Patients (*N* = 8520)					
L1 vertebra attenuation* (ΔHU/yr [± SD])	−1.84 [5.22]	−2.48 [6.36]	−0.73 [8.12]	< 0.001	0.007
L3 muscle attenuation* (ΔHU/yr [± SD])	−0.78 [1.23]	−1.08 [1.38]	−1.00 [1.41]	< 0.001	0.002
L3 muscle area (Δcm^2^/yr [± SD])	0.57 [2.46]	−0.20 [2.69]	−0.53 [2.67]	< 0.001	0.023
L3 VAT attenuation* (ΔHU [± SD])	−0.14 [1.20]	−0.03 [1.25]	0.31 [1.28]	< 0.001	0.024
L3 VAT area (Δcm^2^/yr [± SD])	3.31 [8.99]	2.67 [9.12]	0.44 [9.11]	< 0.001	0.019
L3 SAT area (Δcm^2^/yr [± SD])	4.55 [13.89]	2.17 [13.20]	−1.90 [11.54]	< 0.001	0.040
L3 VAT/SAT ratio (Δ/yr [± SD])	0.007 [0.038]	0.008 [0.041]	0.008 [0.045]	0.965	N/A

*Adjusted for IV contrast media

VAT visceral adipose tissue, SAT subcutaneous adipose tissue

Of note, R^2^ values for age as a predictor of rate of change was generally small for all body composition measures for both sexes (range, 0.002–0.040), suggesting that age overall has a small effect on the observed differences.

## Discussion

In this study, AI tools were used to assess body composition measures (L1 vertebral body attenuation, L3 level skeletal muscle attenuation and area, L3 level VAT attenuation and area, L3 level SAT area, and VSR) derived from abdominal CT scans in 15,616 unique patients at two time points at least five years apart. We observed significant age and sex-specific differences in intra-patient rates of body composition change over time. For example, while both sexes show a similar rate of decrease in muscle attenuation (density) across the age spectrum, in middle and old age men show a significantly faster decline of muscle area while women show a significantly faster decline in bone density.

It is now feasible to deploy AI tools on large patient data sets (with prior study patient sample sizes numbering in the tens or hundreds of thousands [6, 7]) with the goal of clinical implementation of these tools for opportunistic screening and risk stratification. Prior works have focused on examining the relationship between body composition, disease, and health outcomes, as well as establishing normative ranges for body composition measures at the population level. Importantly, these prior studies have relied almost entirely on cross-sectional patient samples when examining trends over time.

The current study is novel and adds to prior work by leveraging a large patient sample to examine longitudinal, intra-patient changes in body composition measures across CT examinations performed at two time points. The analysis is strengthened by limiting inclusion to patients whose CT examinations were at least five years apart, with a mean CT interval of 9.1 years and a maximum of 20.4 years. The ability to avoid short examination intervals while maintaining a large sample size allows for reduced noise and increased confidence when determining rates of change. Furthermore, the use of intra-patient measurements allows for observed rates of change to be directly studied.

Examination of rates of change is important because while cross-sectional studies have established that body composition measures change over time it is crucial to understand at what age these measures are likely to be changing the most—potentially allowing for increased attention and intervention at times when the impact is likely to be highest—and whether relevant differences exist between sexes. For example, both sexes experience a decline in vertebral attenuation with age, which has been shown to correlate with fragility fracture risk. However, in this study the greatest decline in vertebral attenuation was in the 18–39 age group for men but the 40–59 age group for women; these observations support findings from prior studies showing the greatest decreases in bone mineral density among women in perimenopause and early menopause [20] and comparatively lower bone mineral density among younger men when compared with younger women [29]. Additionally, declines in muscle attenuation (which have been associated with metabolic syndrome, reductions in physical function, and overall mortality [30–34]) were found to accelerate with increasing age for males but appeared to plateau after middle age in females. These examples suggest that different age- and sex-dependent strategies may be necessary to mitigate risk and optimize outcomes associated with these measures.

Furthermore, the results obtained from the comparatively large patient sample used in this study may provide important context regarding prior body composition studies conducted on more specific patient groups. For example, in a sample of patients who lost weight with semaglutide treatment, Nelson, et al., [11] observed a mean total decrease in L3 level VAT area from 341 to 309 cm^2^ and SAT area from 410 to 371 cm^2^ compared with an expected mean change in VAT area of 0.44–5.96 cm^2^/yr and mean change in SAT area of −1.90-4.55 cm^2^/year depending on age and sex based on the current study. Similarly, a number of the studies included in the meta-analysis of body composition in the setting of Crohn’s disease by Tang, et al., [12] use L3 level skeletal muscle measurements as a marker of sarcopenia; the mean values for muscle attenuation and area reported in this study could serve as useful reference points.

This study has several limitations. There may be selection bias which limits generalizability of results as data were retrospectively gathered from patients presenting for CT examinations as a part of medical care, and specific CT indication was not considered. These patients, especially inpatients, may carry a greater burden of overall disease than the population in general. This limitation should in part be mitigated by the large number of patients in the study, broad inclusion criteria, and inclusion of multiple clinical settings (inpatient, outpatient, emergent, and urgent). Although the study sample was large, data were gathered from a single Midwestern medical center, which may limit generalizability to other populations. There was no attempt to perform subgroup analysis by race, ethnicity, or socioeconomic factors. The study inclusion criteria required patients to present for imaging at the same institution at two time points at least five years apart; patients unable to meet these criteria for any reason (e.g., relocation, change of insurance, transportation or other access issues, etc.) would have been excluded from the study sample. Finally, the relationship between changes in body composition over time and comorbid diseases and health outcomes were beyond the scope of this manuscript but certainly represent an area for potential future study.

In conclusion, longitudinal intra-patient changes over time in CT-based composition measures were generally found to be significantly different between male and female patients. In addition, certain biomarkers showed different rates of change amongst younger, middle-age, and older adults. These findings suggest that varying age- and sex-dependent strategies may help mitigate risk and optimize health outcomes associated with these measures and could allow for better resource allocation where impact may be greatest.

## Data Availability

The data that support these findings are not in an openly available format but may be provided upon reasonable request.

## Abbreviations

AI: Artificial intelligence
HU: Hounsfield units
SAT: Subcutaneous adipose tissue
SD: Standard deviation
VAT: Visceral adipose tissue
VSR: VAT/SAT ratio
